# A case of apical ballooning syndrome in a male with status asthmaticus; highlighting the role of B2 agonists in the pathophysiology of a reversible cardiomyopathy

**DOI:** 10.3402/jchimp.v3i2.20530

**Published:** 2013-07-05

**Authors:** Farah F. Salahuddin, Peter Sloane, Philip Buescher, Lev Agarunov, Divya Sreeramoju

**Affiliations:** 1 Department of Medicine, Medstar Union Memorial Hospital, Baltimore, MD, USA; 2 Pulmonary Critical Care, Medstar Union Memorial Hospital, Baltimore, MD, USA; 3 Department of Medicine, American University of Antigua, New York, NY, USA

**Keywords:** pulmonary critical care, cardiology, medicine

## Abstract

Apical ballooning syndrome (ABS), also known as Takotsubo cardiomyopathy, was first reported by Dote and colleagues in Japanese literature in 1991 in a review of five cases. Case series have highlighted the association of severe psychological stressors as the major precipitating factors of this syndrome. Status Epilepticus and Sub-Arachnoid hemorrhage are also now established independent etiologies for this phenomenon in patients without coronary artery disease. We report a case of reversible apical ventricular dysfunction in a 50-year-old male presenting with status asthmaticus who quickly underwent intubation. Following this, he had ST elevations in precordial leads with mild cardiac enzyme leak. Subsequent cardiac catheterization revealed a left ventricular ejection fraction of 25–30% with apical aneurismal segment. No obstructive disease was observed. Three days later there was marked clinical improvement; the patient was extubated and repeat echocardiography revealed a remarkable return to normal ventricular size and systolic function. Our case demonstrates that excess use of beta-agonists may be a potential risk factor for ABS and raises the possibility of cathecholamine cardiotoxicity being mediated via beta-receptors. Furthermore, it also negates the propensity of apical ballooning so far reported only in women with respiratory distress without confounding emotional stressors.

Takotsubo cardiomyopathy (also referred to as apical ballooning syndrome [ABS] or stress cardiomyopathy) is a reversible dysfunction of the left ventricle that has been attributed to a rapid surge of endogenous catecholamines brought on by severe emotional or physical stress (1). Recently there have been a few cases of Takotsubo cardiomyopathy occurring secondary to exogenous catecholamine administration, such as epinephrine or norepinephrine, when used as a medical intervention (2, 3). However, in this case, we raise the possibility of Takotsubo cardiomyopathy in a male patient, that has been brought on by the excessive use of inhaled beta-2-agonists at home. Though our understanding of ABS remains relatively theoretical, the nature of this case leads us to the consideration that the potentially life-threatening neurohumoral effect on the myocardium can be induced by the prolonged and excessive use of adrenaline-like substances, such as inhaled beta-2-agonists. Such a consideration brings forth ABS as a differential worth medical investigation when an asthmatic patient presents with congestive heart failure in the face of acute respiratory failure.

## Case report

A 50-year-old man with asthma since childhood presented to the emergency room with 7 days history of asthma exacerbation and acute respiratory distress, requiring immediate intubation and mechanical ventilation. His family had noticed escalating use of inhalers by the patient over the preceding 2 weeks. The patient had used albuterol sulfate metered dose inhaler and albuterol nebulizer every 2–3 hours daily for the preceding week without clinical improvement. During the last 24 hours prior to emergency admission, he was using albuterol sulfate metered dose inhaler 2 puffs (90 µg each) and 3 mL (2.5 mg) of inhalation solution in a nebulizer at least every 2 hours, perhaps as frequently as every 15 min. His pharmacist confirmed that he had consumed a 1-month supply of both inhaler and nebulizer forms of albuterol sulfate in less than 1 week.

The patient had a medical history of hypertension and alcohol abuse. There was no personal or family history of cardiac disease, diabetes, or hyperlipidemia. The family confirmed that he was under no unusual emotional stress at the time of presentation. The patient had a history of prior severe asthma exacerbations requiring emergency room visits and hospital admissions and though he did not smoke at the time of admission, he had a prior 30-pack year history of tobacco use.

Physical examination immediately after intubation and mechanical ventilation showed a temperature of 36.7°C; heart rate of 102 beats/min; blood pressure of 237/109 mmHg, and respiratory rate of 16 breaths per min with oxygen saturation 98%. Auscultation of the chest revealed bilateral wheezes and rhonchi. The general examination was otherwise unremarkable.

White blood cell count was 15.8 K/µL, hemoglobin was 15.6 gm/dL, glucose was 172 mg/dL, and toxicology was negative for cocaine and tricyclics. The cardiac enzymes peaked at 16 hours (creatine phosphokinase, 198 U/L; troponin-I, 2.29 ng/mL; creatine kinase MB, 7.82 ng/mL).

**Fig. 1:**
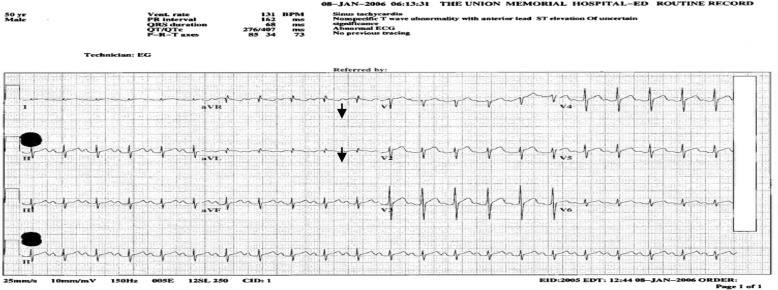
EKG done in the ER showing sinus tachycardia and ST elevations.

**Fig. 2:**
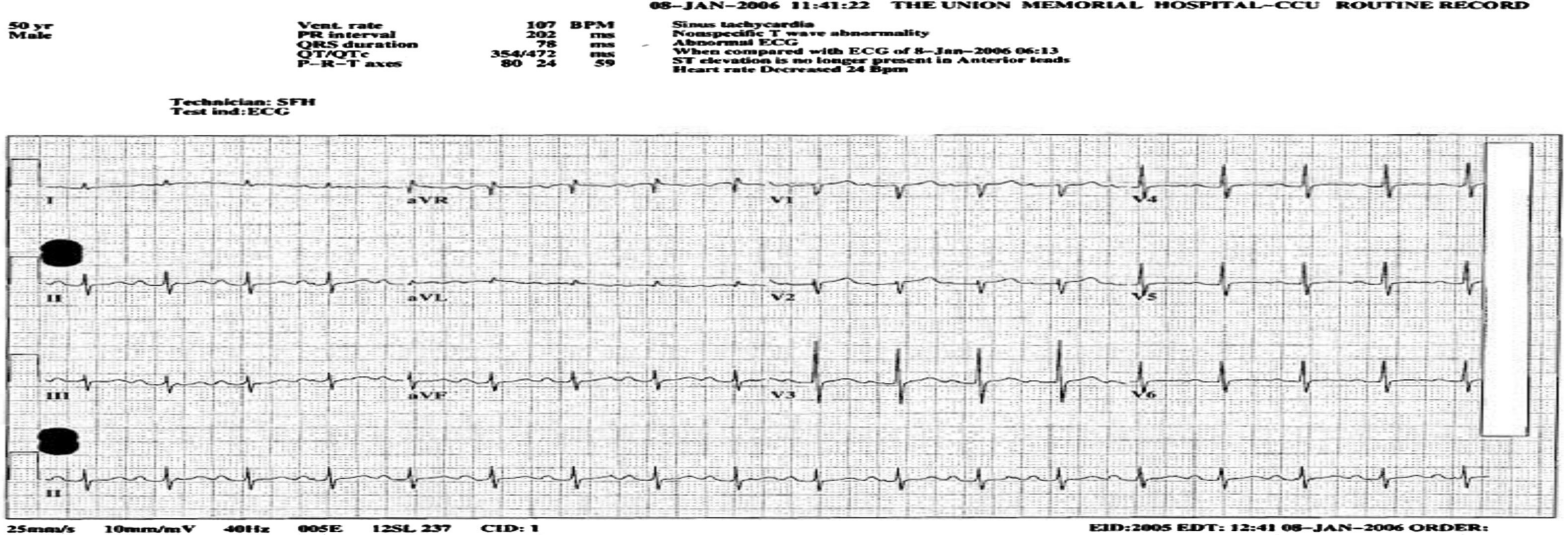
EKG done 5 hours later showing reversal of aforementioned abnormalities.

**Fig. 3:**
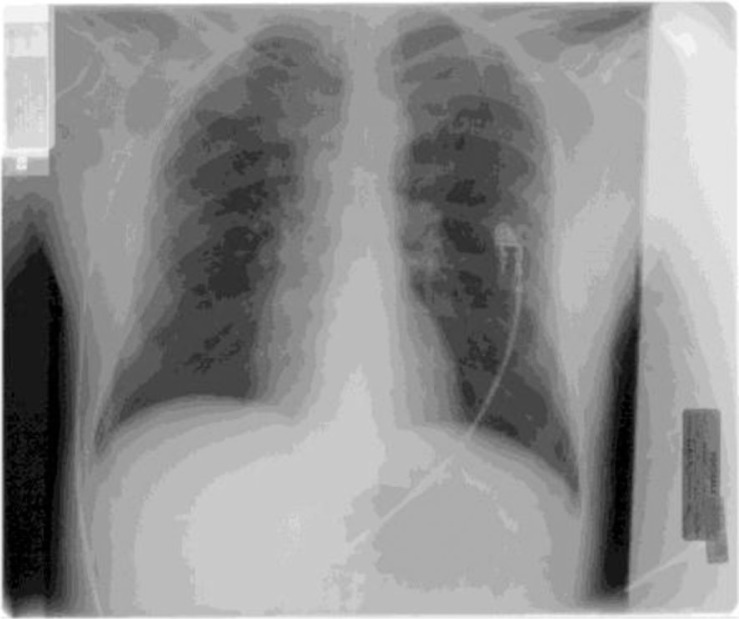
Chest radiograph showing no underlying infiltrates or cardiomegaly.

EKG at the time of admission showed sinus tachycardia with anterior ST elevations in the precordial leads and corrected QT interval of 407 ms (Fig. 1). Follow-up EKG obtained 5 hours after admission showed resolution of anterior ST elevation (Fig. 2). Chest radiograph showed clear lung fields with no infiltrates or cardiomegaly (Fig. 3).

**Fig. 4:**
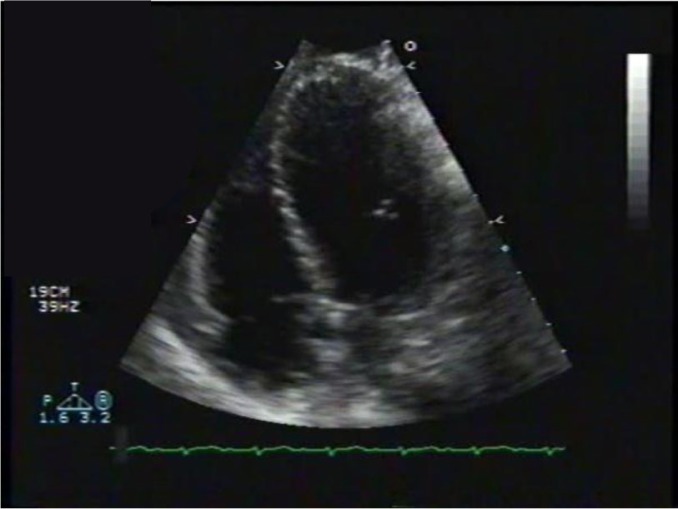
Echocardiogram, pre-diastolic.

**Fig. 5:**
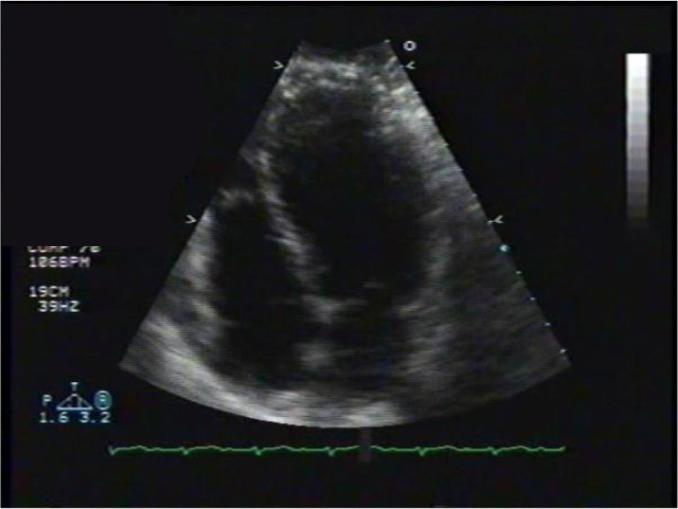
Echo, Pre-systolic.

**Fig. 6:**
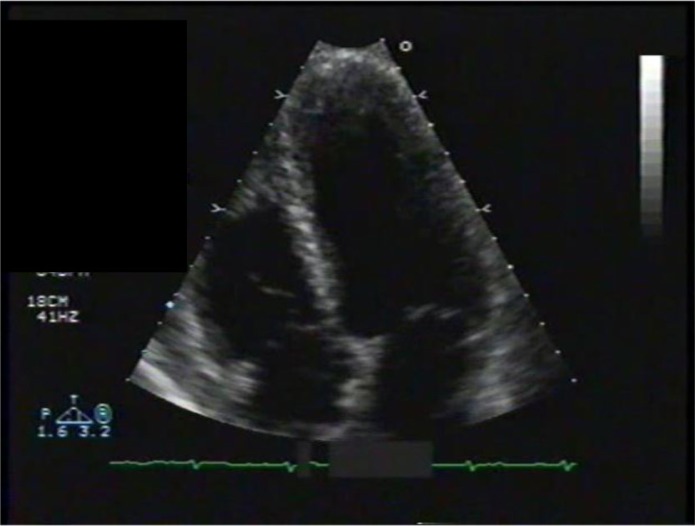
Echocardiogram, post-diastolic.

**Fig. 7:**
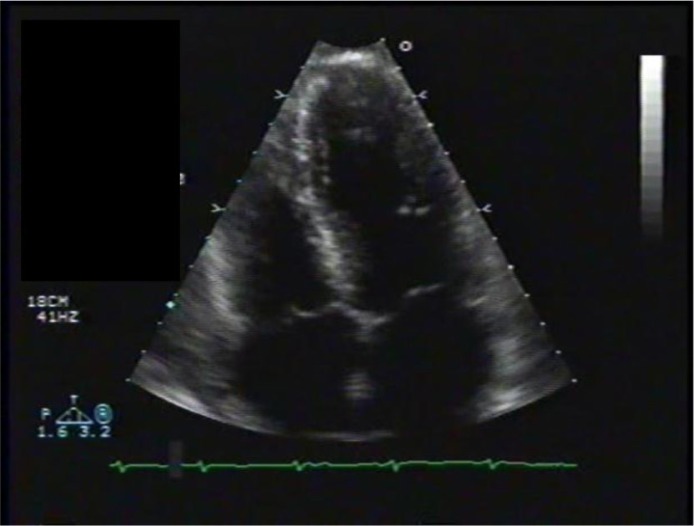
Echocardiogram, post-systolic. Note that there is not much difference between the post-systolic and the pre-systolic images suggesting some stunning of the myocardium.

**Fig. 8:**
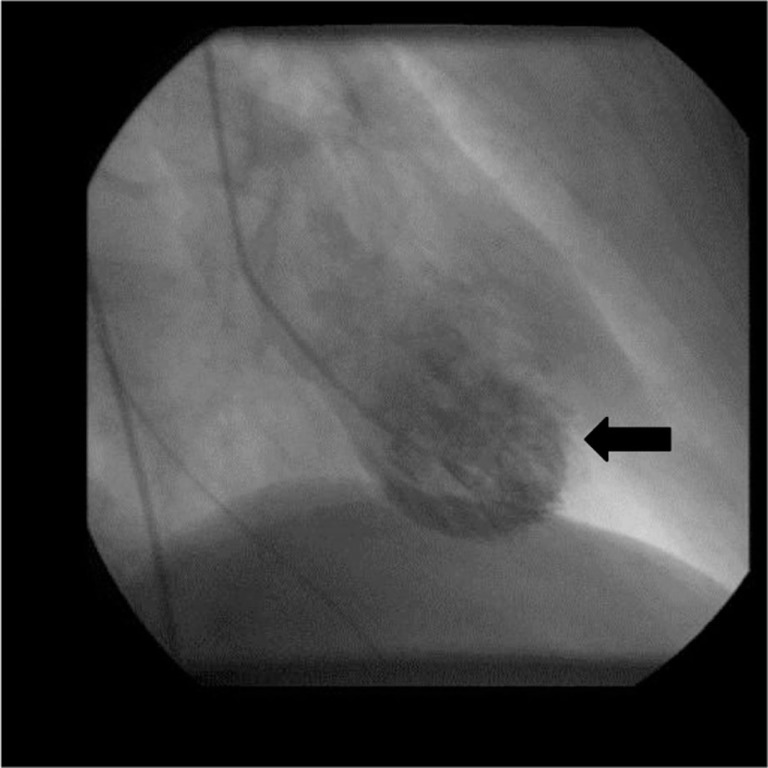
Cardiac catheterization showing apical ballooning in the left ventricle.

Transthoracic echocardiography showed poor left ventricular systolic function, a marked anterior focal wall motion abnormality in the distribution of the left anterior descending artery, and mild-to-moderate tricuspid regurgitation (Figs. 4–7). Emergency cardiac catheterization revealed clinically insignificant luminal irregularities in the left anterior descending coronary artery. Left ventriculography showed an ejection fraction of 25–30% with a large aneurysmal segment in the anteroapical, apical, and inferoapical walls. The inferobasal and anterobasal segments had preserved wall motion. (Fig. 8)

Three days after admission, the patient was weaned and extubated from mechanical ventilation and remained comfortable on room air without resting or exertional dyspnea; a repeat transthoracic echocardiogram showed normal left ventricular function (with resolution of prior wall motion abnormalities) and normalization of the ejection fraction to 60–65%. Seven days after admission the patient was discharged home on montelukast, inhaled salmeterol, fluticasone, tiotropium, and a tapering course of prednisone. He was advised to limit the use of beta-2-agonists to eight puffs a day. A 3-month follow-up showed the patient doing well with no clinical evidence of a recurrence of left ventricular dysfunction.

## Discussion

Apical ballooning syndrome (ABS), also known as Takotsubo cardiomyopathy, was first reported by Dote and colleagues in the Japanese literature in 1991 in a review of five cases (4). More recent case series have highlighted the association of severe psychological stressors as the major precipitating factors in this syndrome (5, 1). It has been hypothesized that elevated catecholamine levels from extreme psychological distress contribute to myocardial stunning by inducing ischemia, possibly from epicardial coronary arterial spasm or by direct myocyte injury. However, the relative importance of these contributing factors to the syndrome of apical ballooning is not clear.

Patients with ABS may present with acute chest pain, pulmonary edema, or cardiogenic shock. The electrocardiogram in this syndrome typically demonstrates ST segment elevation, diffuse T-wave inversions, and abnormal QRS morphology. Echocardiography, coronary angiography, and left ventriculography typically reveal marked apical aneurysmal ventricular dilatation and wall motion abnormalities with no significant coronary artery disease. The myocardial enzyme release into the blood is small relative to the extent of myocardial dysfunction seen on echocardiography or left ventriculography (1). The findings in our patient were consistent with these EKG and enzyme leak patterns.

In this report, we offer a case of ABS in the absence of a strong emotional trigger classically associated with this syndrome. We hypothesize that ABS in our patient was most likely induced by the incessant use of inhaled albuterol sulfate. His total dose of albuterol sulfate was greater than 50 mg per day (maximum recommended dose in adults is 32 mg per day). In our patient, a combination of endogenous elevation of catecholamines from physical distress and exogenous administration of beta-agonist may have precipitated ABS.

To our knowledge, this is only the second reported case of beta-2-agonist-induced ABS in the absence of strong emotional distress, the first being a recurrent case in a 76-year-old female reported in June 2012 (6). Kurisu et al. in 2005 reported high levels of circulating norepinephrine in a female patient as a result of inhaled salbutamol, but the patient also had a recent family death, a classic trigger of ABS (7) and until very recently the most significant risks reported with nebulized and oral beta-2-agonist use have been cardiovascular death, ischemic heart disease, and cardiac failure, but not ABS (8). Very recently, two other cases of ABS have been reported in status asthmaticus in which the development of apical ballooning was reported as a sequela of inhaled beta-2-agonists, but both cases were observed in females (6, 9). Most cases of ABS are observed in post-menopausal women (9) where sensitivity to raised catecholamine levels may be higher (1), leaving this case to be considered a rare presentation.

It is impossible to be certain that exogenous catecholamines from excessive use of beta-agonists were the proximate cause of ABS in this patient rather than secondarily elevated entirely from endogenous catecholamine release related to the stress of his acute medical illness. In spite of this contingency, in one of the other aforementioned cases, the patient repeatedly developed ABS after incessant use of her inhaled beta-2-agonists at home with no other emotional or physical stressors involved in any of the episodes (6). Given this stronger correlation, the possibility of beta-2-agonists being the primary cause of this patient's ventricular dysfunction can be appreciated to a much more significant extent (10–12)

Diagnosis: Acute respiratory failure due to a combination of status asthmaticus and congestive heart failure from acute ABS (Takotsubo cardiomyopathy syndrome).

## Pearls

1. Exogenous catecholamines may precipitate ABS via cardiotoxicity mediated by beta-receptors in distinction to more classic causes, where ABS may be mediated by endogenous catecholamine release in the setting of severe psychological stress.
2. To the best of our knowledge, ABS has previously been observed only in females in status asthmaticus. This phenomenon may not be limited to females, as shown in our case.
3. In patients with asthma when respiratory failure presents atypically with congestive heart failure, clinicians should elicit a history of excessive use of beta-agonists and consider obtaining cardiac enzymes with echocardiography to exclude ABS.
